# Precision pharmacology in menopause: advances, challenges, and future innovations for personalized management

**DOI:** 10.3389/frph.2025.1694240

**Published:** 2025-11-13

**Authors:** Zhi-qing Guo

**Affiliations:** Xiamen Hospital of Traditional Chinese Medicine, Xiamen, Fujian, China

**Keywords:** menopause, hormone replacement therapy (HRT), non-hormonal therapies, vasomotor symptoms (VMS), genitourinary syndrome of menopause (GSM), osteoporosis, precision medicine, emerging therapies

## Abstract

Menopause, characterized by ovarian function cessation and estrogen decline, affects over a billion women globally, leading to vasomotor symptoms (VMS), genitourinary syndrome of menopause (GSM), mood disturbances, osteoporosis, and cardiovascular risks. Pharmacological management is essential for symptom alleviation and long-term health, yet debates on hormone therapy risks necessitate personalized approaches. This review synthesizes recent advances in menopause pharmacology, evaluating hormonal therapies, non-hormonal alternatives, emerging options, challenges, and future directions. A systematic literature search, following PRISMA guidelines, was conducted via PubMed, Cochrane Library, and Web of Science (2015–2025) using keywords like “menopause pharmacological therapy,” “hormone replacement risks,” “non-hormonal VMS treatments,” and “ovarian aging modulators.” Two independent reviewers screened abstracts and full texts, including RCTs, meta-analyses, and expert consensuses focused on efficacy, safety, pharmacokinetics, and mechanisms; exclusions applied to non-English or pre-2015 studies. Hormonal therapies (MHT/HRT), evolved from WHI trials, effectively reduce VMS by 70%–90% and preserve bone density via estrogen receptor modulation, with low-dose transdermal regimens minimizing VTE and breast cancer risks per NAMS/IMS guidelines. Non-hormonal options like SSRIs/SNRIs (40%–60% efficacy) and NK3R antagonists (fezolinetant, 50%–65% VMS reduction) suit contraindicated patients. Emerging therapies, including phytoestrogens, testosterone for libido, and ovarian aging modulators (e.g., AMH analogs), address unmet needs. Special populations (e.g., POI, cancer survivors) require tailored strategies, while challenges include access inequities and long-term data gaps. Advancements underscore precision pharmacology's shift to individualized, non-hormonal treatments. Future priorities: biomarker-guided personalization, AI-driven discovery, and novel delivery systems to enhance efficacy, reduce risks, and improve QoL for menopausal women.

## 1 Introduction

Menopause represents a pivotal physiological transition in women's lives, defined as the permanent cessation of menstruation due to ovarian follicular depletion, typically occurring around the age of 51 years in Western populations (1). This phase is characterized by a decline in endogenous estrogen and progesterone levels, leading to dysregulation of the hypothalamic-pituitary-ovarian axis and a cascade of systemic changes (2). Globally, menopause affects over a billion women, with projections indicating that by 2025, more than 1.1 billion women will have entered this stage, underscoring its profound public health implications (3). From a pharmacological perspective, menopause is not merely a reproductive endpoint but a window for targeted interventions to mitigate associated symptoms and long-term health risks.

The clinical burden of menopause is multifaceted, encompassing vasomotor symptoms (VMS) such as hot flashes and night sweats, which afflict up to 80% of women, as well as genitourinary syndrome of menopause (GSM), mood disturbances, sleep disruptions, and increased susceptibility to osteoporosis and cardiovascular disease (4, 5). These manifestations significantly impair quality of life, productivity, and healthcare utilization, with economic costs estimated in the billions annually (6). Pharmacological management is essential, as lifestyle modifications alone often prove insufficient for moderate-to-severe cases, particularly in women with comorbidities that preclude certain therapies (7). Moreover, the transition amplifies risks for chronic conditions; for instance, estrogen deficiency accelerates bone resorption, elevating fracture risk by 50%–100% in the decade post-menopause (8).

Contemporary pharmacological strategies for menopause management have evolved considerably, with menopausal hormone therapy (MHT) remaining the cornerstone for symptom alleviation in appropriate candidates (9, 10). MHT, typically involving estrogen alone or combined with progestogens, acts via estrogen receptor modulation to restore hormonal balance, effectively reducing VMS by 70%–90% and preserving bone mineral density (11, 12). Recent guidelines from the North American Menopause Society (NAMS) and International Menopause Society (IMS) endorse low-dose, individualized regimens initiated within 10 years of menopause onset or before age 60, emphasizing benefits like cardiovascular protection and fracture prevention while acknowledging risks such as venous thromboembolism and breast cancer in prolonged use (13). The “timing hypothesis” posits that early intervention maximizes benefits and minimizes harms, supported by reanalyses of landmark trials like the Women's Health Initiative (WHI) (10).

For women contraindicated for MHT—such as those with breast cancer history or thrombotic risks—non-hormonal alternatives have gained prominence (14). Selective serotonin reuptake inhibitors (SSRIs) like paroxetine and serotonin-norepinephrine reuptake inhibitors (SNRIs) such as venlafaxine modulate neurotransmitter pathways to alleviate VMS, with efficacy rates of 40%–60% (14). A breakthrough in this domain is fezolinetant, a neurokinin-3 receptor (NK3R) antagonist approved by the FDA in 2023, which targets hypothalamic KNDy neurons to reduce VMS frequency by over 50% without hormonal effects (15). Emerging options include phytoestrogens, gabapentinoids, and testosterone for libido enhancement, though evidence varies in robustness (12).

Despite these advances, significant gaps persist that limit optimal menopause management. For instance, long-term safety data on non-hormonal agents remain sparse, with most trials spanning only 12–24 months, raising uncertainties about sustained efficacy and rare adverse effects (4). Inter-individual variability in treatment response, influenced by genetic polymorphisms (e.g., CYP2D6 for SSRIs) and comorbidities, lacks robust biomarkers for prediction, hindering precision approaches (14). Moreover, clinical trials often underrepresent diverse ethnic groups and low-resource settings, perpetuating inequities in access and outcomes, compounded by gaps in provider education on menopause symptoms (10). These unknowns underscore the need for integrated, evidence-based strategies to enhance personalization and equity.

This review aims to address these gaps by synthesizing recent pharmacological evidence for menopause management, evaluating risks and benefits across therapies, and proposing future directions such as biomarker-guided personalization and novel drug delivery systems to optimize outcomes and enhance women's health during this critical life stage.

## 2 Methods

This narrative review adopted elements from systematic review methodologies to reduce selection bias and ensure a robust bibliographic strategy, as recommended for enhancing narrative reviews (16). A comprehensive literature search was conducted from January 2015 to October 2025 using PubMed (Bethesda, Maryland, USA; accessing MEDLINE, PubMed Central, and other NCBI databases), Cochrane Library (London, UK), and Web of Science (Philadelphia, Pennsylvania, USA; Clarivate). Search terms included “menopause pharmacological therapy,” “hormone replacement therapy risks benefits,” “non-hormonal menopause treatments,” “vasomotor symptoms pharmacology,” “ovarian aging modulators,” and related variants, combined with Boolean operators (AND/OR) for precision.

**Figure 1:**
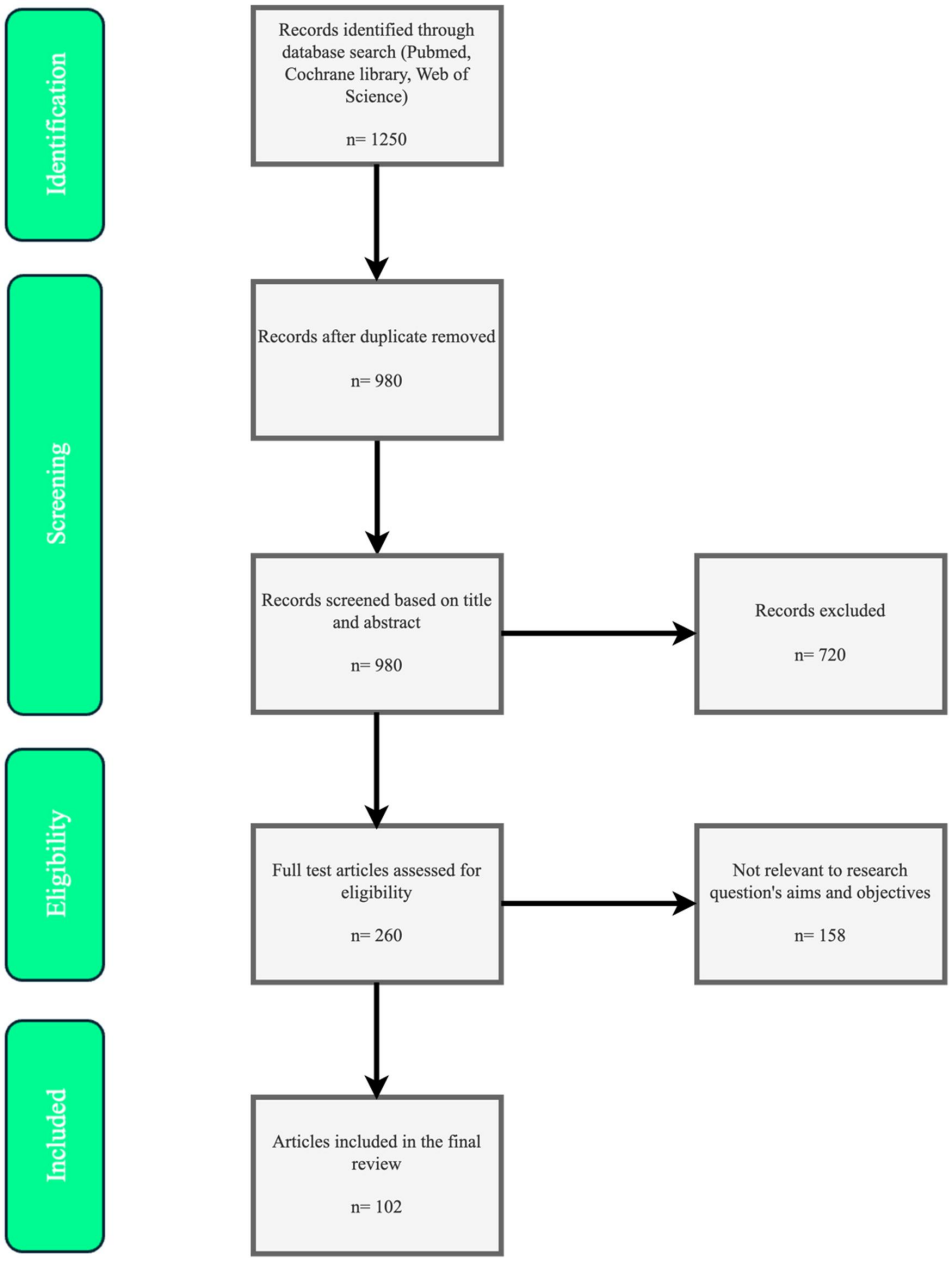
PRISMA-inspired flow diagram for literature selection.

Inclusion criteria emphasized English-language peer-reviewed studies, including randomized controlled trials (RCTs), meta-analyses, systematic reviews, and expert guidelines/consensuses focused on pharmacological mechanisms, efficacy, safety, pharmacokinetics, and clinical outcomes. Exclusions applied to non-English articles, pre-2015 publications (except foundational studies like WHI for context), case reports, editorials, or unrelated topics. This approach aimed to provide a balanced, evidence-based synthesis while minimizing subjectivity in article selection, as shown in Figure 1.

The search results yielded evidence clustered into key analyzed topics, which form the structure of this review: (1) Physiological mechanisms of menopause and pharmacological targets, covering ovarian senescence and systemic impacts; (2) Hormonal therapies: evolution, evidence, and guidelines, including MHT refinements from WHI; (3) Non-hormonal pharmacological options, such as SSRIs/SNRIs and NK3R antagonists; (4) Emerging and adjunctive therapies, like phytoestrogens and ovarian aging modulators; (5) Management in special populations, addressing POI, cancer survivors, and metabolic conditions.

### 2.1 Physiological mechanisms of menopause and pharmacological targets

Menopause is fundamentally driven by the progressive depletion of ovarian follicles, leading to a decline in ovarian steroid hormone production, particularly estradiol (E2) and progesterone (1). This process, often termed ovarian senescence, begins in the perimenopausal phase with irregular menstrual cycles and culminates in the permanent cessation of menses after 12 consecutive months of amenorrhea. At the cellular level, ovarian aging involves hallmarks such as genomic instability, telomere attrition, epigenetic alterations, and impaired proteostasis, which collectively reduce follicular viability and responsiveness to gonadotropins (17). Epigenetic factors, including DNA methylation and histone modifications, play a crucial role in modulating gene expression related to reproductive health, influenced by environmental exposures like endocrine disruptors (18). As ovarian function wanes, serum levels of follicle-stimulating hormone (FSH) rise due to diminished inhibin B feedback, while luteinizing hormone (LH) pulses become more erratic, reflecting dysregulation of the hypothalamic-pituitary-ovarian (HPO) axis (1, 19).

The HPO axis serves as the central regulator of reproductive hormones, where gonadotropin-releasing hormone (GnRH) from the hypothalamus stimulates pituitary FSH and LH secretion, which in turn promote ovarian steroidogenesis (1). In menopause, the loss of negative feedback from ovarian steroids and inhibins leads to hypergonadotropism, exacerbating symptoms and long-term health risks (17). A key mediator in this axis is the kisspeptin/neurokinin B/dynorphin (KNDy) neuron system in the arcuate nucleus of the hypothalamus. KNDy neurons co-express kisspeptin (a potent GnRH stimulator), neurokinin B (NKB, which enhances neuronal excitability via NK3 receptors), and dynorphin (an inhibitory opioid). During menopause, estrogen withdrawal causes hypertrophy and hyperactivity of KNDy neurons, leading to pulsatile GnRH release and contributing to vasomotor symptoms (VMS) like hot flashes (1). This hyperactivity is a prime pharmacological target, as antagonizing NK3 receptors can normalize thermoregulatory disruptions without hormonal supplementation.

Beyond reproductive hormones, menopause induces systemic physiological changes through estrogen deficiency, affecting multiple organ systems (20). Vasomotor symptoms arise from altered thermoregulation in the hypothalamus, where reduced estrogen levels lower the threshold for heat dissipation, involving neurotransmitters like serotonin (5-HT) and norepinephrine (NE). Genitourinary syndrome of menopause (GSM) results from atrophy of estrogen-sensitive tissues in the vulva, vagina, and lower urinary tract, leading to dryness, dyspareunia, and urinary issues due to decreased collagen and vascularity (1). Bone metabolism is profoundly impacted; estrogen normally inhibits receptor activator of nuclear factor kappa-B ligand (RANKL) while promoting osteoprotegerin (OPG), maintaining bone homeostasis (21, 22). Post-menopause, increased RANKL/OPG ratio accelerates osteoclast activity, resulting in rapid bone loss and osteoporosis risk (22). Cardiovascular effects stem from endothelial dysfunction, as estrogen receptors (ER*α* and ER*β*) mediate vasodilation and anti-inflammatory actions; their decline promotes atherosclerosis and hypertension (20, 23). Cognitive and mood changes are linked to estrogen's neuroprotective roles, including modulation of synaptic plasticity via brain-derived neurotrophic factor (BDNF) and GABAergic/serotonergic pathways, potentially increasing vulnerability to depression and cognitive decline (20, 24).

These mechanisms highlight several pharmacological targets for menopause management. Estrogen receptors (ERs), particularly ER*α* in bone and cardiovascular tissues and ER*β* in the brain and urogenital tract, are primary sites for hormone replacement therapies (HRT), which mimic endogenous E2 to restore signaling (1). Selective estrogen receptor modulators (SERMs) like raloxifene target ERs tissue-specifically, offering bone protection without uterine stimulation (22). The NK3 receptor on KNDy neurons represents a novel non-hormonal target; antagonists such as fezolinetant block NKB signaling, reducing VMS by stabilizing GnRH pulsatility (1). Neurotransmitter systems provide additional avenues: selective serotonin reuptake inhibitors (SSRIs) and serotonin-norepinephrine reuptake inhibitors (SNRIs) enhance 5-HT and NE availability to mitigate VMS and mood symptoms (24). GABAergic agents like gabapentin modulate neuronal excitability for symptom relief (1). For bone health, bisphosphonates and denosumab target RANKL pathways to inhibit osteoclasts, while anabolic agents like parathyroid hormone analogs stimulate osteoblasts (21, 22). Emerging targets include epigenetic modifiers to delay ovarian aging and anti-inflammatory agents to address chronic low-grade inflammation (“inflammaging”) associated with menopause (17, 18). Phytoestrogens, such as isoflavones from soy, act as weak ER agonists, potentially alleviating symptoms through dietary modulation (25).

Understanding these mechanisms enables precision pharmacology, tailoring interventions to individual profiles, such as genetic polymorphisms in ER genes or metabolic status (18). Future research should focus on integrating multi-omics data to identify novel targets, enhancing therapeutic efficacy while minimizing adverse effects.

### 2.2 Hormonal therapies: evolution, evidence, and guidelines

Hormonal therapies, often referred to as menopausal hormone therapy (MHT) or hormone replacement therapy (HRT), have undergone significant evolution since their inception in the mid-20th century. Initially introduced in the 1940s with conjugated equine estrogens (CEE) for alleviating menopausal symptoms, MHT gained widespread popularity in the 1960s and 1970s as a means to combat aging and maintain femininity, with prescriptions peaking in the 1990s. The landscape shifted dramatically in 2002 with the early termination of the estrogen-plus-progestin arm of the Women's Health Initiative (WHI) trial, a large-scale randomized controlled trial (RCT) involving over 16,000 postmenopausal women, which reported increased risks of coronary heart disease (CHD), stroke, pulmonary embolism, and breast cancer associated with combined CEE and medroxyprogesterone acetate (MPA). This led to a precipitous decline in MHT use, dropping from approximately 17.9 million prescriptions in the US in 2002 to 3.7 million by 2013, as fears amplified by media coverage deterred both clinicians and patients (26). Subsequent reanalyses of WHI data, however, nuanced these findings, revealing that risks were age-dependent and formulation-specific, prompting a reevaluation and resurgence in tailored MHT approaches (12, 27). By the 2020s, MHT has evolved toward lower doses, transdermal routes, and bioidentical hormones, reflecting a more personalized paradigm informed by long-term follow-up studies and updated guidelines (9, 13).

The mechanisms underlying MHT involve restoring hormonal balance through exogenous estrogens and, when necessary, progestogens to mimic premenopausal physiology. Estrogens, primarily 17*β*-estradiol (E2), bind to estrogen receptors (ER*α* and ER*β*) distributed across tissues, exerting genomic effects via nuclear transcription and non-genomic actions through membrane-bound receptors, influencing vasodilation, bone remodeling, and neuronal function (12). Progestogens, such as micronized progesterone or synthetic progestins like MPA, are added to oppose estrogen's proliferative effects on the endometrium in women with intact uteri, reducing hyperplasia risk via progesterone receptor modulation (13). Delivery modalities include systemic (oral, transdermal patches/gels, or implants) for widespread symptoms like vasomotor symptoms (VMS) and osteoporosis prevention, and local (vaginal creams, rings, or tablets) for genitourinary syndrome of menopause (GSM) with minimal systemic absorption (11). Bioidentical hormones, chemically identical to endogenous ones (e.g., micronized E2 and progesterone), have gained favor over synthetic formulations like CEE due to potentially favorable pharmacokinetics, including reduced hepatic first-pass metabolism in transdermal forms, which lowers thrombotic risks by avoiding increased clotting factors (28). Recent innovations include tissue-selective estrogen complex (TSEC) like bazedoxifene with CEE, combining estrogen's benefits with selective estrogen receptor modulator (SERM) protection against endometrial stimulation (9).

Evidence from RCTs and meta-analyses underscores MHT's efficacy while highlighting a nuanced risk-benefit profile. For VMS, MHT achieves 70%–90% reduction in frequency and severity, surpassing non-hormonal alternatives, as demonstrated in the WHI and subsequent trials like the Kronos Early Estrogen Prevention Study (KEEPS), which showed low-dose oral or transdermal E2 improving hot flashes without adverse cognitive effects in recently menopausal women (10, 11). In GSM, local estrogens restore vaginal epithelium, increasing lubrication and pH balance, with systematic reviews reporting significant improvements in dyspareunia and urinary symptoms (11). Bone protection is robust; MHT reduces fracture risk by 20%–40%, preserving bone mineral density (BMD) via ER-mediated inhibition of osteoclastogenesis, as evidenced by WHI follow-up data showing sustained benefits post-discontinuation in younger users (12). Cardiovascular outcomes depend on timing: the “timing hypothesis” posits that MHT initiated within 10 years of menopause or before age 60 confers cardioprotection by maintaining endothelial integrity, supported by WHI reanalyses indicating reduced CHD risk (HR 0.52–0.76) in women aged 50–59, contrasted with increased risks in older cohorts due to plaque destabilization (10, 27). Cancer risks are formulation-specific; combined MHT slightly elevates breast cancer incidence (additional 4–6 cases per 1,000 women over 5 years), per WHI and meta-analyses, but estrogen-alone therapy may reduce it (HR 0.77), and bioidentical regimens show lower associations (27). Venous thromboembolism (VTE) risk is higher with oral routes (RR 1.5–2.0) than transdermal (RR ∼1.0), attributable to hepatic effects on coagulation (29). Long-term WHI extensions (up to 20 years) confirm no overall mortality increase, with benefits outweighing risks for symptomatic women under 60 (27).

Contemporary guidelines reflect this evidence, advocating individualized MHT. The 2022 North American Menopause Society (NAMS) Position Statement designates MHT as first-line for moderate-to-severe VMS and GSM in women without contraindications, recommending low-dose, short-duration use (typically <5 years) initiated early post-menopause, with annual risk reassessment. NAMS endorses transdermal routes for VTE risk reduction and bioidenticals for potentially better tolerability, while cautioning against compounded hormones due to unregulated dosing (11). The International Menopause Society (IMS) 2022–2025 recommendations align, emphasizing MHT's role in preventing osteoporosis and potential cognitive benefits when started perimenopausally, with shared decision-making incorporating absolute risks (e.g., < 1% increase in breast cancer for most users) (30). The British Menopause Society (BMS) and Women's Health Concern (WHC) 2020/2025 updates extend MHT for premature ovarian insufficiency (POI) until age 51, highlighting benefits for cardiovascular and bone health in this group. The US Preventive Services Task Force (USPSTF) 2022, however, advises against MHT for primary prevention of chronic diseases, focusing on symptom management. Personalization factors include age, time since menopause, baseline risks (e.g., via tools like the Gail model for breast cancer), and preferences, with guidelines urging baseline mammograms and lipid profiles (9, 30).

Despite progress, challenges persist, including underutilization due to lingering WHI misconceptions and disparities in access. Future guidelines may incorporate genomics for predicting response and risks, fostering even more precise MHT strategies. This evolution positions MHT as a safe, effective tool when appropriately prescribed, transitioning seamlessly to discussions on non-hormonal alternatives for contraindicated patients, with key comparisons summarized in Table 1.

**Table 1:** Comparison of menopausal hormone therapy (MHT) formulations: efficacy, risks, and guideline recommendations.

Therapy type	Mechanism/target	Main benefits	Risks	Evidence level	Guideline recommendations	Ref
Oral CEE + MPA (Combined)	ER modulation via oral estrogens and progestins; hepatic first-pass effect	VMS relief (70%–90%); bone protection (20%–40% fracture reduction); GSM improvement	Increased VTE (RR 1.5–2.0); breast cancer (HR 1.26); CHD in older women	RCTs (WHI); meta-analyses	NAMS: First-line for VMS/GSM in <60 years; avoid for CVD prevention; short-term use	(11, 12)
Transdermal E2 (Alone or Combined)	Direct ER*α*/*β* activation; avoids hepatic metabolism	VMS reduction (70%–90%); cardioprotection in early menopause (HR 0.52–0.76 for CHD); bone density preservation	Lower VTE risk (RR ∼1.0); minimal breast cancer increase in short use	RCTs (KEEPS, WHI reanalysis); long-term follow-up	IMS/NAMS: Preferred for VTE risk; initiate within 10 years of menopause; annual reassessment	(10, 97)
Bioidentical E2 + Progesterone	Mimics endogenous hormones; ER/PR modulation	Similar to synthetic but better tolerability; VMS/GSM relief; potential lower cancer associations	VTE similar to oral if not transdermal; endometrial hyperplasia if unbalanced	Meta-analyses; observational studies	BMS: Endorsed for symptomatic women; monitor lipids/mammograms; until age 51 for POI	(13, 98)
TSEC (Bazedoxifene + CEE)	Tissue-selective ER agonism/antagonism	VMS relief; bone protection without progestin; reduced endometrial stimulation	Mild GI upset; no VTE increase noted	Phase III RCTs; FDA-approved	NAMS: Alternative for intact uterus; not for CVD prevention; short-duration	(11)

### 2.3 Non-hormonal pharmacological options

Non-hormonal pharmacological options have become increasingly vital in menopause management, particularly for women contraindicated for menopausal hormone therapy (MHT) due to conditions such as breast cancer, thromboembolic disorders, or personal preferences to avoid hormones (14, 31). These therapies primarily target vasomotor symptoms (VMS), such as hot flashes and night sweats, which affect up to 80% of menopausal women and significantly impair quality of life (32). Unlike MHT, non-hormonal agents do not restore estrogen levels but modulate neurotransmitter pathways, thermoregulatory centers, or other physiological mechanisms implicated in menopausal symptoms (33). Recent advancements, including FDA approvals and updated guidelines from 2020 to 2025, have expanded the evidence base, emphasizing efficacy, tolerability, and patient-centered selection (32, 34). This section reviews key classes, including selective serotonin reuptake inhibitors (SSRIs) and serotonin-norepinephrine reuptake inhibitors (SNRIs), neurokinin-3 receptor (NK3R) antagonists, gabapentinoids, and other agents, focusing on mechanisms, clinical evidence, and guidelines.

#### 2.3.1 Selective serotonin reuptake inhibitors (SSRIs) and serotonin-norepinephrine reuptake inhibitors (SNRIs)

SSRIs and SNRIs represent a cornerstone of non-hormonal therapy, repurposed from their primary antidepressant roles to address VMS through central nervous system modulation (31). These agents enhance serotonin (5-HT) and norepinephrine (NE) availability in the brain, stabilizing thermoregulatory centers in the hypothalamus disrupted by estrogen withdrawal (33). Paroxetine mesylate (7.5 mg daily), the only FDA-approved SSRI for VMS, reduces hot flash frequency by 40%–60% in RCTs, with meta-analyses confirming superiority over placebo (mean reduction: 5–7 episodes/week) (14, 31). Venlafaxine (37.5–75 mg extended-release), an SNRI, demonstrates similar efficacy, with studies showing 50%–60% symptom reduction and improved sleep quality, though side effects like nausea and dry mouth affect 20%–30% of users (32). Escitalopram and desvenlafaxine also show promise, with 2023–2025 reviews highlighting their role in women with comorbid mood disorders, where dual benefits on VMS and depression are observed (33). Pharmacokinetics favor low doses to minimize cytochrome P450 interactions, particularly in patients on tamoxifen, where paroxetine's CYP2D6 inhibition is contraindicated (31). Long-term data (up to 12 months) indicate sustained efficacy with low discontinuation rates, but guidelines recommend monitoring for sexual dysfunction and weight gain (14, 32).

#### 2.3.2 Neurokinin-3 receptor (NK3R) antagonists

A breakthrough in non-hormonal therapy is fezolinetant, an NK3R antagonist approved by the FDA in 2023 for moderate-to-severe VMS (34). This agent targets the KNDy neuron hyperactivity in the hypothalamus, where neurokinin B (NKB) signaling exacerbates thermodysregulation post-estrogen decline (33). Phase III trials (SKYLIGHT 1 and 2) demonstrated 50%–65% reduction in VMS frequency at 45 mg daily, with rapid onset (within 1 week) and sustained effects over 52 weeks, outperforming placebo without hormonal risks (34). Safety profiles are favorable, with mild headaches and gastrointestinal upset in <10% of participants, and no significant liver toxicity in post-marketing surveillance as of 2025 (31, 33). Compared to SSRIs/SNRIs, fezolinetant offers hormone-free specificity, making it ideal for breast cancer survivors, as endorsed in 2023 NAMS guidelines (32). Emerging NK3R agents like elinzanetant are in late-stage trials, showing similar efficacy with potential dual benefits for sleep disturbances.

#### 2.3.3 Gabapentinoids

Gabapentinoids, including gabapentin and pregabalin, provide another option by modulating calcium channels and GABAergic transmission to dampen neuronal excitability linked to VMS (31). Gabapentin (900–2,400 mg/day, titrated) reduces hot flashes by 45%–60% in RCTs, particularly effective for nocturnal symptoms, with meta-analyses supporting its use in women intolerant to SSRIs (14). Side effects like dizziness and somnolence limit adherence (20%–30% dropout), but extended-release formulations improve tolerability. Pregabalin (150–300 mg/day) shows comparable efficacy with fewer CNS effects, though evidence is sparser (35). These agents are particularly useful in patients with neuropathic pain comorbidities, but guidelines caution against long-term use due to dependency risks (32).

#### 2.3.4 Other agents

Other pharmacological agents include clonidine, an alpha-2 adrenergic agonist, and oxybutynin, an anticholinergic (14, 32). Clonidine (0.1 mg transdermal patch weekly) modestly reduces VMS (20%–40%) by central sympatholytic effects but is limited by hypotension and dry mouth (31). Oxybutynin (5–15 mg extended-release) achieves 50%–70% symptom relief via muscarinic receptor blockade, with recent trials (2020–2024) highlighting its efficacy in refractory cases, though urinary retention is a concern (33). For genitourinary syndrome of menopause (GSM), ospemifene (60 mg daily), a SERM, improves vaginal health without systemic estrogen risks, reducing dyspareunia in RCTs, as per AUA/SUFU guidelines (36).

Guidelines from 2020 to 2025, including the 2023 NAMS Nonhormone Therapy Position Statement, recommend SSRIs/SNRIs or fezolinetant as first-line for VMS in MHT-contraindicated women, with gabapentin as second-line (10, 32). The British Menopause Society (BMS) 2025 consensus emphasizes evidence-based selection, noting limited efficacy of phytoestrogens and supplements (37). Challenges include variable response rates (30%–70%) and lack of long-term data beyond 2 years, with calls for head-to-head trials (33). Non-hormonal options bridge gaps in care, paving the way for emerging therapies like neuromodulators and personalized approaches, as detailed in Table 2.

**Table 2:** Summary of Non-hormonal pharmacological options for menopause management: mechanisms, efficacy, and safety.

Drug class/example	Mechanism	Efficacy	Side effects/safety	Approval/evidence	Guideline recommendations	Ref
SSRIs (e.g., Paroxetine 7.5 mg)	5-HT reuptake inhibition; thermoregulatory stabilization	VMS reduction 40%–60%; mood benefits in comorbid depression	Nausea, sexual dysfunction (20%–30%); CYP2D6 interactions	FDA-approved for VMS; RCTs/meta-analyses	NAMS/BMS: First-line for MHT-contraindicated; monitor in tamoxifen users	(14, 32, 35)
SNRIs (e.g., Venlafaxine 37.5–75 mg ER)	5-HT/NE reuptake; hypothalamic modulation	VMS relief 50%–60%; improved sleep	Dry mouth, nausea; withdrawal risk	Off-label; RCTs (e.g., 12-month data)	NAMS: Alternative to SSRIs; preferred in mood disorders; low discontinuation	(14, 99)
NK3R Antagonists (e.g., Fezolinetant 45 mg)	Blocks NKB on KNDy neurons; reduces GnRH pulsatility	VMS frequency down 50%–65%; rapid onset (1 week)	Headaches/GI upset (<10%); no hormonal risks	FDA 2023; Phase III (SKYLIGHT trials)	NAMS 2023: First-line for breast cancer survivors; sustained 52-week efficacy	(14, 32, 100)
Gabapentinoids (e.g., Gabapentin 900–2,400 mg)	Ca2 + channel modulation; GABAergic neuronal dampening	Nocturnal VMS reduction 45–60%	Dizziness/somnolence (20%–30% dropout); dependency potential	Off-label; meta-analyses	NAMS: Second-line for SSRI-intolerant; useful in neuropathic pain	(14, 32)
Other (e.g., Clonidine 0.1 mg patch; Oxybutynin 5–15 mg ER)	Alpha-2 agonism (Clonidine); muscarinic blockade (Oxybutynin)	Modest VMS relief (20%–70%); refractory cases	Hypotension/dry mouth (Clonidine); urinary retention (Oxybutynin)	Off-label; recent RCTs (2020–2024)	BMS: Limited use; monitor BP/bladder function; adjunctive options	(14, 35, 99)

### 2.4 Emerging and adjunctive therapies

Emerging and adjunctive therapies for menopause represent a dynamic frontier in pharmacology, aiming to address unmet needs such as persistent symptoms in MHT-contraindicated patients, long-term health maintenance, and delaying ovarian aging. These approaches include (1) plant-derived compounds,(2) selective modulators, (3) androgen supplementation, (4) novel experimental drugs, and (5) nutritional adjuncts, often used in combination with established treatments (3, 30). Recent innovations from 2023 to 2025 emphasize precision medicine, with a shift toward non-hormonal, targeted agents that minimize risks while enhancing efficacy. For instance, advancements in neuromodulators and tissue-selective complexes offer alternatives for vasomotor symptoms (VMS) and genitourinary syndrome of menopause (GSM), while research into ovarian rejuvenation explores anti-Müllerian hormone (AMH) analogs to extend reproductive lifespan (38). Adjunctive therapies, including supplements, support symptomatic relief and preventive care, particularly for bone and cardiovascular health. This section evaluates these options, focusing on mechanisms, clinical evidence, and integration into menopause management.

#### 2.4.1 Plant-derived compounds and selective modulators

Phytoestrogens, naturally occurring plant compounds with estrogen-like activity, and selective estrogen receptor modulators (SERMs) form a key category of adjunctive therapies. Phytoestrogens, such as isoflavones from soy (e.g., genistein and daidzein), act as weak ER agonists or antagonists, binding preferentially to ER*β* to modulate estrogen signaling without the potency of endogenous estradiol (39). This tissue-selective action may alleviate VMS, improve bone density, and support cardiovascular health by reducing lipid peroxidation and inflammation. A 2023 systematic review and meta-analysis of randomized controlled trials (RCTs) involving postmenopausal women found no significant effects on estrogenicity measures (e.g., endometrial thickness or hormone levels) with soy isoflavones, suggesting safety but limited efficacy for severe symptoms (25). However, some studies indicate modest VMS reduction (20%–30%) in Asian populations with higher baseline phytoestrogen intake, potentially due to gut microbiome variations in equol production (40, 41). SERMs like bazedoxifene, often combined with conjugated estrogens in tissue-selective estrogen complexes (TSECs), provide bone protection via ER*α* agonism in skeletal tissue while antagonizing ER in the breast and uterus, reducing hyperplasia risk (42). The FDA-approved Duavee (bazedoxifene/CEE) has shown promise in preventing invasive breast cancer, with 2025 ASCO data from multicenter trials indicating reduced incidence in high-risk postmenopausal women (43). Evidence supports their adjunctive use for osteoporosis prevention, with meta-analyses reporting 15%–25% BMD improvement, though long-term cancer data remain under scrutiny (44).

#### 2.4.2 Androgen supplementation

Testosterone therapy emerges as a targeted adjunct for addressing hypoactive sexual desire disorder (HSDD) and related symptoms in menopausal women. Endogenous testosterone declines gradually with age, contributing to reduced libido, energy, and muscle mass via androgen receptor (AR) modulation in the brain, genitalia, and skeletal muscle (45, 46). Transdermal testosterone (off-label, 150–300 μg daily) enhances sexual function by increasing arousal and orgasm frequency through dopaminergic and nitric oxide pathways, without significantly raising serum levels beyond premenopausal ranges (47). A 2019–2024 review of nearly 8,500 women demonstrated significant HSDD improvement, with 2024 RCTs confirming mood and cognitive benefits, including reduced fatigue and enhanced vulvovaginal health (48, 49). Guidelines from the British Menopause Society (BMS) and International Menopause Society (IMS) endorse testosterone for postmenopausal HSDD unresponsive to other interventions, recommending monitoring for androgenic side effects like acne or hirsutism (incidence <5%) (50–52). Challenges include lack of FDA-approved formulations for women, leading to compounded products, and concerns over long-term cardiovascular risks, though 2023–2025 data suggest neutrality when doses are physiologic.

#### 2.4.3 Novel experimental drugs

Novel directions in menopause pharmacology focus on delaying ovarian aging and innovative delivery systems. AMH analogs, such as those developed by Celmatix, activate the AMH receptor (AMHR2) to suppress primordial follicle recruitment, potentially preserving ovarian reserve and extending menopause onset by 5–10 years (53). Preclinical studies (2017–2024) show agonist analogs reducing follicle loss in chemotherapy models, with 2025 phase I trials exploring applications in premature ovarian insufficiency (POI) (54). Other experimental agents target autophagy and telomerase to mitigate genomic instability in oocytes, with pharmacological strategies like rapamycin analogs showing promise in animal models for extending fertility (54). Nano-delivery systems, such as liposomal estradiol or targeted nanoparticles, enhance bioavailability and reduce systemic exposure, minimizing risks like VTE; early 2024 trials report improved GSM relief with vaginal nano-formulations (55, 56). Combination therapies, integrating NK3R antagonists with SERMs or androgens, are under investigation for potential synergistic effects on VMS and sexual function (57).

#### 2.4.4 Nutritional adjuncts

Nutritional supplements serve as adjuncts, bolstering pharmacological interventions. Vitamin D, often combined with calcium, supports bone health by enhancing calcium absorption and modulating parathyroid hormone, crucial in estrogen-deficient states (58). RCTs from 2019 to 2024 show 1,000-2,000 IU daily reducing fracture risk by 15%–20% in at-risk postmenopausal women, with 2024 Endocrine Society consensus advising RDA dosing for healthy adults and targeted supplementation for deficient (>20 ng/ml) or high-risk individuals (59). Omega-3 fatty acids (e.g., EPA/DHA 1–2 g daily) exert anti-inflammatory effects via prostaglandin modulation, potentially alleviating joint pain; however, meta-analyses indicate no significant overall benefit for VMS, with only exploratory subgroup signals (e.g., in obesity) (60–62). Evidence in breast cancer survivors supports their role in mitigating aromatase inhibitor side effects (63). Guidelines advocate these as safe adjuncts, though interactions with anticoagulants warrant caution.

These emerging and adjunctive therapies expand the pharmacological toolkit, promoting individualized care. Future research should prioritize RCTs and real-world evidence to validate long-term outcomes, transitioning to special populations in subsequent discussions.

### 2.5 Management in special populations

Menopause management requires tailoring to special populations, where physiological, pathological, or sociodemographic factors alter symptom presentation, risks, and therapeutic responses. These groups include women with (1) premature ovarian insufficiency (POI), (2) surgical menopause, (3) cancer survivors, those with (4) metabolic conditions like obesity or diabetes, and (5) individuals with racial or ethnic variations in symptom profiles. Pharmacological strategies must prioritize safety, efficacy, and personalization, incorporating guidelines from 2020 to 2025 that emphasize evidence-based adjustments to mitigate accelerated health risks such as cardiovascular disease (CVD), osteoporosis, and reduced quality of life (QoL) (5). Genomic insights, such as CYP2D6 polymorphisms, further enable precision approaches to optimize drug metabolism and outcomes.

#### 2.5.1 Premature ovarian insufficiency (POI) and surgical menopause

Women with POI or surgical menopause (e.g., post-bilateral oophorectomy) experience abrupt estrogen deficiency, accelerating risks for CVD, bone loss, and cognitive decline, often before age 40 (64–66). Guidelines recommend menopausal hormone therapy (MHT) until the average age of natural menopause (approximately 51 years) to prevent morbidity, using physiological doses of transdermal estradiol (50–100 μg/day) combined with micronized progesterone (100–200 mg/day cyclically) for endometrial protection in non-hysterectomized women (5, 67, 68). This approach reduces fracture risk by 30%–50% and improves vasomotor symptoms (VMS) and QoL, as supported by 2024–2025 ASRM and ESHRE guidelines. For those with contraindications, non-hormonal options like SSRIs or NK3R antagonists (e.g., fezolinetant) address VMS, while bisphosphonates or denosumab manage osteoporosis (68). Emerging therapies, including melatonin or stem cell interventions, show promise in preclinical studies for restoring ovarian function, though clinical data remain limited (68, 69). Lifestyle integration, such as diet and exercise, enhances outcomes, with 2025 reviews highlighting their role in symptom mitigation (69).

#### 2.5.2 Cancer survivors

Cancer survivors, particularly those with breast or endometrial cancer, face heightened challenges due to estrogen-sensitive tumors, precluding systemic MHT (70, 71). For breast cancer survivors, non-hormonal therapies are prioritized; acupuncture and cognitive behavioral therapy (CBT) reduce hot flashes by 40%–60%, per 2020–2024 meta-analyses, while SSRIs/SNRIs (e.g., venlafaxine 75 mg/day) offer symptomatic relief without increasing recurrence risk (70, 72). Local vaginal estrogens (e.g., low-dose estradiol cream) are considered safe for genitourinary syndrome of menopause (GSM) in most cases, with 2024 Lancet reviews indicating minimal systemic absorption and no elevated cancer risk (73). Ospemifene, a SERM, provides an alternative for GSM, improving vaginal health in RCTs without promoting breast tumor growth (71). For tamoxifen users, avoiding CYP2D6 inhibitors like paroxetine is crucial to prevent reduced efficacy (74). Overall, multidisciplinary care, including oncologist input, is essential, with non-estrogenic options like fezolinetant emerging as game-changers for VMS.

#### 2.5.3 Women with metabolic conditions (e.g., obesity or diabetes)

In women with obesity or type 2 diabetes mellitus (T2DM), menopause exacerbates insulin resistance and weight gain, increasing CVD and metabolic risks (75, 76). MHT, particularly oral estrogens, may improve glucose homeostasis and delay T2DM onset by 15%–20%, but transdermal routes are preferred to avoid hepatic effects in obese patients (75). GLP-1 receptor agonists like tirzepatide (combined with MHT) facilitate significant weight loss (10%–15%) and glycemic control, as per 2025 Endocrine Society data (77, 78). Metformin remains first-line for T2DM management, with adjunctive benefits on VMS, while lifestyle interventions (e.g., caloric restriction) are foundational (79, 80). Guidelines advocate monitoring for VTE in obese women on MHT, favoring low-dose regimens.

#### 2.5.4 Racial and ethnic variations

Racial and ethnic differences influence menopause experiences; Black and Hispanic women often enter menopause earlier (by 6–12 months) and report more severe VMS, vaginal dryness, and sleep disturbances compared to White women (81). Asian women may experience fewer hot flashes but more somatic symptoms, potentially due to dietary phytoestrogens or genetic factors (82). Structural racism and socioeconomic disparities exacerbate these, with Black women facing higher CVD risks and lower MHT utilization (83). Culturally sensitive pharmacology, including equitable access to non-hormonal options, is recommended, with 2024 studies highlighting underprescription in minority groups (81).

Personalized pharmacology integrates genomics; CYP2D6 polymorphisms affect metabolism of SSRIs and tamoxifen, with poor metabolizers (PMs) requiring alternative therapies to avoid inefficacy or toxicity (84). CPIC guidelines endorse genotyping for tamoxifen, recommending aromatase inhibitors for PMs, and similar adjustments for menopausal drugs (85). Sex-specific pharmacogenomics, including hormonal-genetic interactions, supports tailored dosing (86).

Addressing these populations demands holistic, equitable strategies, bridging to broader challenges in menopause care.

### 2.6 Challenges, gaps, and future directions

Despite significant advancements in the pharmacological management of menopause, several challenges persist that hinder optimal care for women worldwide. One major issue is the underutilization of effective therapies, driven by lingering misconceptions from early interpretations of trials like the Women's Health Initiative (WHI), which initially overstated risks of menopausal hormone therapy (MHT) such as breast cancer and cardiovascular events (87). This has led to a decline in MHT prescriptions, with recent surveys indicating that only 4%–10% of symptomatic postmenopausal women in the US receive it, despite its proven efficacy for vasomotor symptoms (VMS) and osteoporosis prevention (88). Adherence remains low, with discontinuation rates exceeding 50% within the first year due to side effects, fear of risks, and inadequate patient education (89). Economic burdens exacerbate this, as menopause-related symptoms contribute to billions in lost productivity and healthcare costs annually, yet access to affordable treatments is limited in low- and middle-income countries (LMICs), where only 10%–20% of women receive any form of management. Global disparities are stark, with inconsistent care, diagnostic delays, and a lack of standardized protocols amplifying inequities, particularly in regions affected by socioeconomic factors or post-COVID-19 healthcare disruptions. The COVID-19 pandemic further compounded challenges by limiting access to consultations and increasing mental health burdens intertwined with menopausal symptoms (20).

Controversies surrounding MHT continue to fuel debates, particularly regarding long-term risks. Recent 2024 analyses reaffirm elevated risks of heart disease and venous thromboembolism with oral estrogen-progestin combinations, while tibolone shows associations with breast and endometrial cancer, underscoring the need for formulation-specific evaluations (90). These risks are age- and duration-dependent, yet public and provider misconceptions persist, deterring utilization even in low-risk groups (9). Non-hormonal therapies, while safer for contraindicated populations, face scrutiny over variable efficacy; for instance, SSRIs achieve only 40%–60% VMS reduction, and long-term data beyond 2 years are sparse, raising questions about sustained benefits vs. placebo effects (1, 44). Emerging agents like NK3R antagonists (e.g., fezolinetant) offer promise but are criticized for high costs and limited accessibility, potentially widening global gaps (3).

Significant knowledge gaps impede progress. Evidence on non-hormonal therapies' long-term safety, such as potential psychiatric or cognitive impacts from SSRIs in older women, remains insufficient, with most RCTs limited to 12–24 months (1, 20). Diversity in research is lacking; trials often underrepresent ethnic minorities, LMIC populations, and comorbidities like obesity, leading to biased guidelines that fail to address inter-individual variability (91). Biomarker development is nascent, with limited validation for predictors like anti-Müllerian hormone (AMH) in guiding therapy personalization (92, 93). The full spectrum of menopausal symptoms' impact on future health—such as links to cardiovascular, psychiatric, and longevity outcomes—is underexplored, with scoping reviews highlighting associations but calling for mechanistic studies (20). Provider knowledge gaps persist, with surveys showing inconsistent education on menopause, resulting in undertreatment and stigma around symptoms like sexual dysfunction (89).

Looking ahead, future directions in menopause pharmacology emphasize innovation and precision. Artificial intelligence (AI) and machine learning are poised to revolutionize drug discovery and personalization; for example, AI-derived models using retinal age gaps as biomarkers could predict reproductive aging and tailor interventions, with 2025 studies demonstrating their utility in ovarian cancer diagnostics that may extend to menopause (93). AI algorithms could analyze multi-omics data to identify novel targets, accelerating development of therapies like calorie restriction mimetics or autophagy inducers to delay ovarian aging (54). Biomarkers such as microRNAs (miRNAs), epigenetic markers, or metabolic profiles offer potential for guiding therapy; recent work on retinal age gaps and ovarian reserve indicators like AMH could enable early preventive strategies (92–94). Extending ovarian function emerges as a transformative goal, with repurposed drugs like rapamycin showing promise in slowing follicle depletion and delaying menopause by 5–10 years in preclinical and early clinical trials (e.g., VIBRANT study) (92). This could mitigate age-related diseases, as ovaries influence systemic aging beyond reproduction. Novel delivery systems, including nanotechnology for targeted hormone release, and combination therapies (e.g., MHT with GLP-1 agonists for metabolic benefits) are under exploration to enhance efficacy and reduce side effects (3).

To bridge gaps, research priorities include multi-center, diverse RCTs with long-term follow-up, real-world evidence from registries, and interdisciplinary studies integrating AI for predictive modeling (1, 92). Global collaborations, as advocated in 2023–2025 toolkits, should focus on LMICs to ensure equitable advancements (6). By addressing these challenges and harnessing emerging technologies, menopause pharmacology can evolve toward preventive, personalized paradigms that enhance women's healthspan and QoL.

## 3 Conclusion

The pharmacological management of menopause has evolved significantly, transitioning from a predominantly hormone-centric approach to a multifaceted, personalized paradigm that incorporates both hormonal and non-hormonal therapies tailored to individual needs, risks, and preferences. Summarizing the main results in order: (1) Physiological mechanisms reveal ovarian senescence and HPO axis dysregulation as key targets, with estrogen deficiency driving systemic changes like VMS and bone loss, enabling interventions via ERs, NK3Rs, and neurotransmitter systems (1, 20, 22). (2) Hormonal therapies, evolved from WHI with low-dose transdermal options, achieve 70%–90% VMS relief and 20%–40% fracture reduction but require age/timing-based risk assessment per NAMS/IMS guidelines (27). (3) Non-hormonal options like SSRIs/SNRIs (40%–60% efficacy) and NK3R antagonists (50%–65% VMS reduction) offer safe alternatives for contraindicated patients, with FDA approvals emphasizing tolerability (57, 95). (4) Emerging therapies expand options through phytoestrogens/SERMs for modest symptom relief, testosterone for HSDD improvement, and experimental drugs like AMH analogs for ovarian delay, supported by preclinical/early trials (96). (5) Special populations benefit from tailored approaches, e.g., MHT until age 51 for POI, non-hormonals for cancer survivors, and GLP-1 agonists with MHT for metabolic conditions, addressing disparities via pharmacogenomics (10, 11, 17, 23, 38). (6) Challenges include underutilization, evidence gaps in long-term data/diversity, and access inequities, while future directions prioritize AI/biomarkers for precision and ovarian extension therapies (2, 6, 15, 23, 24).

From these results, we recommend individualized risk-benefit assessments using guidelines like NAMS 2022/2023, prioritizing transdermal MHT for low-risk women and non-hormonals for others, with pharmacogenomic testing (e.g., CYP2D6) to optimize dosing and avoid interactions. Clinicians should integrate lifestyle adjuncts and monitor annually for adherence. Future research should focus on diverse RCTs, biomarker validation (e.g., miRNAs/retinal age gaps), and innovative deliveries to bridge gaps, ultimately improving QoL and healthspan for menopausal women.
